# Exercise Induced Bronchospasm and associated factors in primary school children: a cross-sectional study

**DOI:** 10.1186/s12887-023-03963-w

**Published:** 2023-04-03

**Authors:** Ogochukwu C. Ofiaeli, Chizalu I. Ndukwu, Nwanneka O. Ugwu, Kenechi O. Nnamani, Joy C. Ebenebe, Ifeoma Egbuonu

**Affiliations:** 1 Department of Paediatrics, Nnamdi Azikiwe University (NAU), Nnamdi Azikiwe University Teaching Hospital (NAUTH), Nnewi, PMB 5025 Anambra state Nigeria; 2 Department of Paediatrics, Nnamdi Azikiwe University Teaching Hospital (NAUTH), Nnewi, Anambra state Nigeria; 3 Department of Paediatrics, Chukwuemeka Odumegwu Ojukwu University (COOU) / Chukwuemeka Odumegwu Ojukwu University Teaching Hospital (COOUTH), Awka, Anambra state Nigeria

**Keywords:** Childhood, Exercise induced bronchoconstriction, Nigeria

## Abstract

**Background:**

Exercise Induced Bronchospasm(EIB) is not equivalent to asthma. As many as 20%of school aged children are estimated to have EIB. In Nigeria, there is still a dearth of information on EIB as a clinical entity.

This study determined the presence of EIB(using pre and post-exercise percentage difference in peak expiratory flow rate(PEFR) and associated factors such as age, gender, social class and nutritional status in primary school children in Nnewi, Anambra state, South-East Nigeria. The study also grouped those with EIB into those with asthma(EIB_A_) and those without asthma(EIB_WA_).

**Methods:**

This was a community based cross-sectional study involving 6–12 year olds. The PEFR was taken at rest and after a 6 min free running test on the school play-ground using a Peak Flow Meter. A diagnosis of EIB was made if there was a decline of ≥ 10%. Those who had EIB were grouped further based on the degree of decline in post-exercise PEFR (a decline ≥ 10% < 25% → Mild EIB, ≥ 25% < 50% → Moderate EIB and ≥ 50% → Severe EIB) and then categorized as those with EIB_WA_/EIB_A_.

**Results:**

EIB in the various minutes post-exercise was as follows: 19.2%(1^st^min), 20.9%(5^th^min), 18.7%(10^th^min), 10%(20^th^min), 0.7%(30^th^min). Mild EIB accounted for the greater proportion in all minutes post-exercise and none of the pupils had severe EIB. Using values obtained in the 5^th^min post-exercise for further analysis, EIB_WA_/EIB_A_ = 84.1%/15.9% respectively. Mean difference in the post-exercise PEFR of EIB/no EIB and EIB_WA_/EIB_A_ was -48.45(t = -7.69, *p* =  < 0.001) and 44.46(t = 3.77, *p* = 0.01) respectively.

Age and gender had a significant association to the presence of EIB and 58% of the pupils with EIB were of high social class. The BMI for age and gender z-scores of all study subjects as well as those with EIB was -0.34 ± 1.21, -0.09 ± 1.09 respectively. Other features of allergy(history of allergic rhinitis: OR–5.832, *p* = 0.001; physical findings suggestive of allergic dermatitis: OR–2.740, *p* = 0.003)were present in pupils diagnosed with EIB.

**Conclusion:**

EIB has a high prevalence in primary school children in Nnewi and the greater proportion of those with EIB had EIB_WA_. EIB therefore needs to be recognized as a clinical entity and stratified properly based on the presence or absence of asthma. This will help the proper management and prognostication.

## Background

Exercise Induced Bronchospasm (EIB) is a condition in which vigorous physical activity triggers acute or transient airway narrowing/spasm in people with heightened airway reactivity [1]. It can be demonstrated by a reduction in the Peak Expiratory Flow Rate (PEFR) which can be measured with a Peak Flow Meter and is a non–invasive procedure [2]. The airway spasm in EIB occurs mostly after and rarely during the exercise and results in a decrease in post–exercise pulmonary function test [1, 3].

The term Exercise Induced Bronchospasm (EIB) is often used interchangeably with Exercise Induced Asthma (EIA) [1, 3–7].However, the term EIA is no longer recommended for use as it may imply incorrectly that exercise causes asthma rather than exacerbate or trigger an attack of asthma [6–8]. Most recent guidelines recommend distinguishing EIB with underlying asthma (EIB_A_) from the occurrence of bronchial obstruction with exercise without other symptoms or signs of asthma (EIB_WA_) [6–8]. This is significant because EIB is a manifestation of bronchial hyper-responsiveness(BHR) and though it may be the first sign of asthma, it can occur in the absence of asthma [6, 7]. The evidence for asthma would include recurring history of respiratory symptoms such as cough, wheeze and shortness of breath in association with variable airflow limitation [9]. The presence of EIB_WA_ also indicates a greater chance of developing asthma in the future and is significant for athletes [6, 7]. EIB may also occur in children with atopy or rhinitis or following respiratory infections [6, 7]. Pathogenesis and management for both EIB_A_ and EIB_WA_ are different [5–8].

Until recently, EIB has not been studied as an independent entity but was used principally as a tool for defining childhood asthma in epidemiological surveys [2, 10–12]. As a result data on EIB as an entity especially with categorization into EIB_A_ and EIB_WA_ is grossly lacking. This is more profound in our environment [5–8].

EIB_WA_ has been reported in school children as well as adults [13]. Most asthmatic patients manifest EIB [13]. It is reported that as many as 90% of asthmatic patients have EIB and this is more frequent in more severe and poorly controlled asthmatics [6–8]. It is therefore reflective of the level of disease control [7, 8].

Exercise Induced Bronchospasm has been widely used to define childhood asthma in epidemiological studies [2, 10–12]. Up to 10% of school children are reported to have EIB [7]. Most recent guidelines however recommend distinguishing exercise induced bronchospasm with underlying clinical asthma – EIB_A_ from occurrence of exercise induced bronchospasm in subjects without other symptoms or signs of asthma – EIB_WA_ [7, 8]. It is now recommended that both should be considered as two separate entities [5, 7, 8].This is because pathogenesis and treatment modalities for both conditions differ [5–8].

A few studies have been carried out on EIB in Nigeria [2, 14, 15]. Majority of these studies used EIB and EIA interchangeably [2, 15]. EIB is a manifestation of bronchial hyperresponsiveness [6, 7].It is often the first sign of asthma but can also occur in the absence of asthma [6, 7]. The presence of EIB_WA_ also indicates a greater chance of developing asthma in the future and is significant for athletes [6]. EIB may also occur in children with atopy or rhinitis or following respiratory infections [7]. Pathogenesis and management for both EIB_A_ and EIB_WA_ are different [5–8].This study hopes to augment the knowledge on both EIB_A_ and EIB_WA_ in Nigeria.

Some studies on airway hyper-responsiveness and asthma have been done in Nigeria and Africa as a whole [2, 10, 11, 16, 17]. These studies while still very relevant did not describe EIB as a clinical entity. The age group of 6—12 year olds was chosen because prevalence of EIB is reported to decrease from childhood to adolescence [11]. In addition, lung function testing, bronchial challenge and other physiological tests do not have a major role in the diagnosis of asthma in children 5 years and younger due to the inability of children of this age group to perform reproducible expiratory maneuvers [18]. Such tests are only possible in specialized centres in that age group [18]. 

Finally, EIB is said to be more frequent and more severe in poorly controlled asthmatic patients [6–8]. Determining the prevalence of EIB_A_ among school children in Nnewi could give insight on how well asthma is being controlled in school children in the environment. Findings could be useful in instituting better awareness of the condition and hopefully bring about better control.

The objectives of this study were to determine the presence of EIB in school children aged 6–12 years; to identify the proportion of primary school children with EIB who have asthma (EIB_A_) and those who do not (EIB_WA_) and to determine the association between nutritional status and socio-demographic characteristics such as age, gender and socio-economic status and the presence of EIB in these children.

## Methods

### Study area

The study was carried out in Nnewi, the second largest city in Anambra state in South-East Nigeria [19]. Geographically, Nnewi falls within the tropical rain forest region of Nigeria [19]. Tropical rain forests can be characterized in two words–hot and wet [20].The main tribe found in Nnewi are the Igbos [19]. The main occupation of Nnewi people is trading and farming [19]. The town is known for its vibrant auto industry and is also home to many major indigenous industries [19]. However, with onset of industrialization in the town around 1970, as well as the presence of a tertiary hospital and medical school in the town, Nnewi is experiencing an influx of other Nigerian tribes and foreigners [19]. Nnewi is a fast developing city and a major industrial and commercial hub in Africa [19].

Population data for primary schools was obtained from the Planning, Research and Statistics (PRS) department of the Nnewi North Zonal offices of the Anambra state Ministry of Education and the State Primary Education Board (SPEB). The schools chosen for the study were distributed among the four autonomous communities in Nnewi.

### Study design

This was a descriptive cross–sectional study involving school children aged 6–12 years in Nnewi, Nnewi North LGA, Anambra state. These are principally primary school children and those enrolled into both public and private primary schools were recruited.

### Subject selection

Apparently well children and children for whom parental consent and child’s assent had been obtained were included in the study. Children who had received oral or inhaled steroids in the previous week, had received a bronchodilator within the preceding 24 h before the exercise challenge or had a baseline PEFR < 70% of predicted were excluded [6–8, 21]. Children with PEFR values less than 70% of expected were excluded from the study because of likely exercise intolerance [6, 7].

### Sample size determination

Minimum sample size was determined with the following formular [22]:

$$\mathrm{n}= \frac{{\mathrm{Z\alpha }}^{2}\mathrm{PQ}}{{\mathrm{d}}^{2}}$$

n is the minimum sample size

Zα is the test statistics for 95% confidence interval = 1.96.

P is the available prevalence of EIB = 40% [23].

Q is 1 – *P* = 1 – 0.4 = 0.6

D is the desired level of precision, 5% = 0.05

$$\begin{array}{ll}\mathrm n&=\frac{{1.96}^2\mathrm x0.4\mathrm x0.6}{{0.05}^2}\\&\begin{array}{cc}=\frac{3.8416\mathrm x0.4\mathrm x0.6}{0.0025}&=368.79\underline\sim369\end{array}\end{array}$$

Anticipating a non-response rate (f) of 20% [24–26],

$$\begin{array}{ccc}\mathrm{Adjusted}\;\mathrm{sample}\;\mathrm{size}\;\left(\mathrm{ns}\right)&=&\frac n{1-\mathrm f}\end{array}$$

$$=\frac{369}{0.8}=461.25\underline\sim461$$

The number of schools selected was calculated by correcting for design effect (deff) using the formula [26, 27]:

$$\begin{array}{ccc}\begin{array}{ccc}\mathrm{Number}\;\mathrm{of}\;\mathrm{schools}&=&\mathrm n\end{array}&\frac xs&\mathrm{deff}\end{array}$$

n is the effective sample size = 369s is the cluster size (number of children to be sampled per school), fixed at 75. [26] deff is the design effect = 2.48 [26].

Therefore,

$$\begin{array}{ccc}\mathrm{Number}\;\mathrm{of}\;\mathrm{schools}&=\frac{392\;\times\;2.48}{75}&=12.2\underline\sim12\;\mathrm{schools}\end{array}$$

### Sampling technique

The Multi-Stage Sampling technique was used both for school selection and recruitment of study subjects. Four public and eight private schools were chosen. Subsequently, simple random sampling using the balloting method was used to select the individual schools included in the study after serially numbering the primary schools in each autonomous community.

Following receipt of ethical approval from the Ethical Review Committee of NAUTH and study permit from the Ministry of Education, Anambra state, the list of students by their classes was obtained from the selected schools. The number of students determined to be recruited per school was then proportionately divided among the classes based on the student population of each class. Final participant recruitment based on the number of students determined to be selected per class was done by simple random sampling using the balloting method. Class registers were used as sampling frames.

### Data collection

A subject information and consent form was given to students to take home to their parents/guardians. Consenting parents/guardians were then given a modified International Study on Allergies and Asthma in Children (ISAAC) [28] self-administered questionnaire to fill. The questionnaire was adapted from phase two and three questionnaires used for the ISAAC study [28].

The height and weight of the children were measured and used to calculate their BMI using the formula weight in kg/height in meters [2]. BMI for age and gender z-scores were then used as the index of nutritional status.

Exercise challenge tests are used for the diagnosis of EIB [29] and the 6 min free running test [2, 11, 12, 21] was used in this study. A peak flow meter was used to obtain the PEFR. Socioeconomic status of the children was determined using Oyedeji’s Social Classification Index [30].

The recruited children were tested in groups of five with a research assistant assigned to each child. The children were taught how to use the metered dose salbutamol inhaler (using a placebo) and peak flow meter prior to the free running test [31, 32]. Quality assurance was ensured by comparing peak flow readings to that of Peak Flow Master Breath-O-Meter brand, made by CIPLA at the onset of the research and at intervals of 100 study subjects.Physical examination was carried out on the study subjects before exercise.

All recruited children were brought down from their classes and made to sit quietly in a designated area for 10 min before the exercise test. Their chest was auscultated by the principal investigator for rhonchi. Other evidence of pre-existing allergic conditions such as features of allergic conjunctivitis [33] and features of allergic dermatitis [28, 34–36] were sought for and documented.

The peak flow reading was taken by asking the child to take his/her deepest breath. While standing and holding the peak flow meter in one hand, the child used the other hand to occlude both nostrils. He/she then wrapped his/her lips around the mouth piece snugly and breathed out as hard and as fast as possible [31, 32]. Nose clips were not used because some of the children found them uncomfortable and refused its use. Three pre exercise PEFRs were obtained and the best (highest) value recorded [2, 29].

The pointer of the peak flow meters were adjusted to zero before each reading. Recruited children were allowed a 6 min free range running exercise challenge on the school playground. This running test was performed usually during their break time (between 10am and 12noon), as permitted by the school authorities. They were instructed to run continuously, as hard as possible so as to achieve maximum intensity within the first 2–3 min and increase pulse rate up to at least 85% maximum for the participant [2, 7, 8, 29, 37].  In order to provide encouragement, the children were cheered while running and on some occasions, the principal investigator/any assistant would participate in parts of the race (mostly at the beginning of the race). A target heart rate calculator was used to confirm that the post exercise increase in heart rate was up to 85% of the resting heart rate [38]. Those children who did not achieve this, repeated the test the following day.

A stop clock and whistle was used to regulate exercise duration. Before each round of exercise, the children were instructed to run when they heard ‘the 1^st^ whistle’ and stop once they heard ‘the 2^nd^ whistle’. The stop clock was always set to zero, started at the onset of a round of exercise and stopped at the end of 6 min. It was used to ensure proper timing of each round of exercise.

PEFR readings were taken as already outlined with the study subjects standing at 1,5,10,20,30 min interval [2, 8, 29, 31, 32]. EIB was diagnosed if the percentage fall in post exercise PEFR ≥ 10% compared to the pre-exercise value [1, 2, 7, 13].

At the end of the exercise, each subject was observed for 30 min for wheeze and other signs of respiratory distress by the principal investigator. Metered dose salbutamol inhalers were available to reverse any symptoms or signs if they arose [2, 6–8]. Spacer devices were also provided. All questionnaires and documented readings were properly coded. Parents/guardians of children diagnosed with EIB were informed, counselled and subsequently referred to the Paediatric Respiratory unit of Nnamdi Azikiwe University Teaching Hospital, Nnewi for further care.

### Data analysis

The Outcome/Dependent variable for this study is categorical and dichotomous – the presence or absence of EIB. This was diagnosed by a ≥ 10% decline in post exercise PEF reading compared with the pre exercise values [1, 2, 7, 13, 29, 39]. The presence of EIB in the 1^st^, 5^th^, 10^th^, 20^th^ and 30^th^ minute was determined. The presence of EIB was analyzed further as a continuous and an ordinal variable having outcomes as follows:

 ≥ 10% < 25% → Mild, ≥ 25% < 50% → Moderate and ≥ 50% → Severe [29]. The time category with the highest prevalence of EIB was then used for further analysis [14].

The Explanatory / Independent variables were:

i. Social – demographic characteristics (which included socio-economic status, age, gender) and nutritional status.
ii. Previous history of atopy – asthma, allergic rhinitis, allergic dermatitis and vernal conjunctivitis.

Nutritional status was analyzed using age and gender z-scores for BMI. Normal is from 1 to -2, > 1 is possible risk of overweight, > 2 is overweight, > 3 is obese while < -2 is wasted and < -3 is severely wasted. The BMI was also analyzed as a continuous variable. Data obtained was analyzed in two groups: EIB in children with no previous history suggestive of asthma (EIB_WA_) and EIB in children with previous history of asthma (EIB_A_) using the statistical package for social sciences (SPSS) version 21 with the assistance of a statistician. EIB with asthma was diagnosed by the presence of at least a history of recurrent wheeze and/ or recurrent cough in the presence of exercise induced bronchospasm [9, 40, 41].

Numerical variables were analyzed as difference between means using the appropriate student t-test. Categorical variables were analyzed using the chi-square (*X* ^2^) analysis for association. Multivariate analysis using logistic regression was also done to determine the independent effect of the explanatory variables on the outcome variable and control for possible confounders. A *p*-value of less than 0.05 was taken to indicate statistical significance.

## Results

### Section A: Results describing the study subjects

This section gives general descriptive results of the study subjects. Data were collected everyday while schools were in session from the 3^rd^ week in February to the 2^nd^ week of April (2^nd^ term of the 2016/2017 school session) during the periods allocated by the school authorities. A total of 470 pupils aged 6–12 years were proportionately sampled from both public and private primary schools. The final number of pupils whose data were analyzed was 422. Reasons for removal from final analysis included severe wasting (27, 56.2%), poorly filled questionnaire (7, 14.6%), non–return of the questionnaire (12, 25.0%) and need for post exercise salbutamol (2, 4.2%).

The age (mean ± SD) of the pupils in this study was 7.75 ± 1.61 years of which 222(52.6%) were males with a M: F ≃ 1.1:1. Their mean body mass index (BMI ± SD) z score was – 0.34 ± 1.21. Majority of the pupils (317, 75.1%) had BMI z score of 1 to -2. Of the total study subjects, 231 (60.4%) were of high social class and 231 (54.7%) were enrolled into private schools. Details of these socio-demographic characteristics are described in Table 1. Among those from the upper class, 88, 34.5% and 167, 65.5% were enrolled into public and private schools respectively.

**Table 1:** Characteristics of the pupils in the study

**Characteristics**	**Frequency (n)**	**Percentage (%)**
**Age (years)**		
** 6**	133	31.5
**7**	83	19.7
**8**	61	14.5
**9**	74	17.5
**10**	51	12.1
**11**	14	3.3
**12**	6	1.4
**Total**	422	100.0
**Gende**r		
**Male/Female**	222/200 (1.1:1)	52.6/47.4
**Socioeconomic class**		
**High**	255	60.4
**Middle**	121	28.7
**Low**	46	10.9
**Religion**		
**Christian**	419	99.3
**Others**	3	0.7
**Tribe**		
**Igbo**	419	99.3
**Others**	3	0.7
**Type of school**		
**Public**	191	45.3
**Private**	231	54.7
**BMI z scores**		
**Normal (1 to -2)**	317	75.1
**Risk of overweight (> 1)**	49	11.6
**Overweight (> 2)**	8	1.9
**Wasted (< -2)**	48	11.4
**Total**	422	100.0

*BMI* Body mass index

The peak expiratory flow rate (PEFR) percentage fall of the subjects (mean ± SD) in the 1^st^ and 5^th^ minutes post – exercise were -3.22 ± 15.86 with minimum/maximum values of -52.94/45.16 and -3.23 ± 16.16 with minimum/maximum values of -43.48/37.50 respectively. Table 2 gives the details of the percentage fall in PEFR in the various durations after the 6 min exercise. PEFR was measured in L/min.

**Table 2:** PEFR Fall (%) following 6 min exercise

**Post–exercise duration (min)**	**EIB/No EIB**	**Frequency**	**Mean percentage fall in post exercise PEFR ± SD**	**Percentage fall in post exercise PEFR**	
				**Minimum**	**Maximum**
**1**	EIB	81	18.00 ± 8.90	10.00	45.16
	No EIB	341	-8.26 ± 12.65	-52.94	9.52
	Total	422	-3.22 ± 15.86	-52.94	45.16
**5**	EIB	88	18.68 ± 7.65	10.00	37.56
	No EIB	334	-9.01 ± 12.44	-43.48	9.09
	Total	422	-3.23 ± 16.16	-43.48	37.50
**10**	EIB	79	19.85 ± 6.90	10.00	31.58
	No EIB	343	-8.60 ± 12.88	-50.00	9.09
	Total	422	-3.27 ± 16.34	-50.00	31.58
**20**	EIB	42	12.90 ± 2.64	10.00	19.05
	No EIB	380	-4.98 ± 10.24	-43.48	8.70
	Total	422	-3.21 ± 11.13	-43.48	19.05
**30**	EIB	3	13.69 ± 1.03	12.50	14.29
	No EIB	419	-4.31 ± 8.98	-47.83	9.38
	Total	422	-4.19 ± 9.08	-47.83	14.29

*EIB* Exercise induced bronchospasm, *SD* Standard deviation

The mean pre – exercise PEFR in the study subjects was 220.43 ± 52.60 L/min with minimum / maximum values of 120 / 370 L/min. The lowest post – exercise peak expiratory flow rate (PEFR) recorded in this study was 110L/min while the maximum was 450L/min. In the 5^th^ min post – exercise, the greatest number of pupils – 88 manifested with EIB having a mean post – exercise PEFR of 187.39 ± 56.31 L/min as against 334 pupils with no EIB and having a mean post – exercise PEFR of 235.84 ± 51.53 respectively. Details of the post – exercise PEFR of the pupils over the various durations after the 6 min exercise are given in Table 3.

**Table 3:** PEFR (L/min) of the pupils in the various durations after 6 min exercise

**Minutes post–exercise(min)**	**EIB/No EIB**	**Frequency**	**Mean post – exercise PEFR ± SD**	**Post – exercise PEFR**	
				**Minimum**	**Maximum**
**1**	EIB	81	195.31 ± 43.79	110	270
	No EIB	341	232.23 ± 55.07	130	450
	Total	422	225.14 ± 55.01	110	450
**5**	EIB	88	187.39 ± 56.31	110	290
	No EIB	334	235.84 ± 51.53	130	390
	Total	422	225.73 ± 56.07	110	390
**10**	EIB	79	173.04 ± 46.83	110	310
	No EIB	343	238.40 ± 53.88	130	450
	Total	422	226.16 ± 58.45	110	450
**20**	EIB	42	194.76 ± 43.52	140	270
	No EIB	380	229.76 ± 53.78	130	440
	Total	422	226.28 ± 53.84	130	440
**30**	EIB	3	213.33 ± 57.74	180	280
	No EIB	419	228.95 ± 54.49	140	450
	Total	422	228.84 ± 54.46	140	450

*EIB* Exercise induced bronchospasm, *SD* Standard deviation

### Section B: Results on the diagnosis of EIB in the study subjects

This section gives results on EIB in the study subjects. It also provides other results supporting this diagnosis. Exercise Induced Bronchospasm (EIB) in the study subjects in the 1^st^, 5^th^, 10^th^, 20^th^ and 30^th^ minutes post exercise are given in Table 4. The 5^th^ minute had the highest prevalence of 20.9% (88).

**Table 4:** Prevalence of Exercise Induced Bronchospasm in the study subjects in the various post exercise durations

**Post exercise duration(min)**	**Presence of EIB**		**Total (%)**
	**Yes (%)**	**No (%)**	
**1**	81 (19.2)	341 (80.8)	422 (100.0)
**5**	88 (20.9)	334 (79.1)	422 (100.0)
**10**	79 (18.7)	343 (81.3)	422 (100.0)
**20**	42 (10.0)	380 (90.0)	422 (100.0)
**30**	3 (0.7)	419 (99.3)	422 (100.0)

Majority of the children had mild EIB with none manifesting with severe EIB (Table 5).

**Table 5:** Prevalence and severity of EIB in the study subjects

**Post exercise duration(min)**	**EIB severity**				Total (%)
	**Mild (%)**	**Moderate (%)**	**Severe (%)**	**No EIB (%)**	
**1**	62 (14.7)	19 (4.5)	_	341 (80.8)	422 (100.0)
**5**	63 (15.0)	25 (5.9)	_	334 (79.1)	422 (100.0)
**10**	53 (12.5)	26 (6.2)	_	343 (81.3)	422 (100.0)
**20**	42 (10.0)	_	_	380 (90.0)	422 (100.0)
**30**	3 ( 0.7)	_	_	419 (99.3)	422 (100.0)

**Fig. 1:**
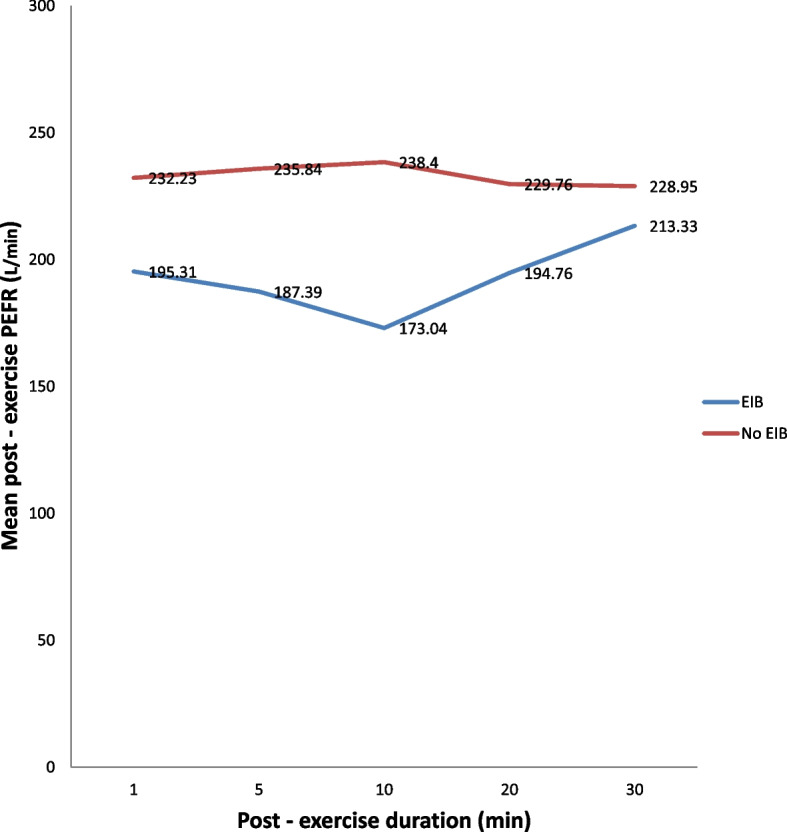
PEFR (L/min) in children with EIB and without EIB following 6 minutes exercise

Figure 1 is a line graph showing the difference in the mean post – exercise PEFR of the children with EIB versus no EIB across the various durations post exercise. There is a wide gap between the mean post – exercise PEFR of children with EIB versus no EIB. Children with EIB had remarkably lower PEF readings.

There was no significant difference in the mean pre-exercise PEFR of children who manifested with EIB versus those with no EIB in the 5^th^ minute post exercise – Table 6.

**Table 6:** Mean pre-exercise PEFR (L/min) of the pupils with EIB versus no EIB

**Post-exercise diagnosis**	**Frequency**	**Mean pre-exercise PEFR**	**Std. Dev**	**T**	***p*** ** – value**	**Mean difference**	**95% CI of the difference**
**EIB**	88	229.20	60.06	1.59	0.114	11.09	- 2.71 to 24.89
**No EIB**	334	218.11	50.29				

*t-test* Test of association, *CI* Confidence interval, *PEFR* Peak expiratory flow rate

### Section C: Results on the diagnosis of EIBWA/EIBA in the study subjects

This section provides results obtained following categorization of pupils with EIB into those without history suggestive of asthma—EIB_WA_ and those with history suggestive of asthma EIB_A_ as well as results supporting the findings. From the foregoing, EIB was highest in the 5^th^ minute post exercise (Tables 4 & 5); the post – exercise PEFRs were also narrowest in the 5^th^ minute post – exercise (Table 3). Consequently, data collected in 5^th^ minute post exercise was used for further analysis (in this section and subsequently). Majority of the children with EIB had EIB_WA_(74, 84.1%) in the 5^th^ minute post exercise – Table 7.

**Table 7:** Categorization of pupils with EIB in the 5^th^ minute post – exercise into those with EIB_WA_ / EIB_A_

**EIB in the 5minute** ^th^	**Frequency (n)**	**Percentage**
	**n**_**1**_ = 422	
**EIB**_**WA**_	74	17.5
**EIB**_**A**_	14	3.3
**No EIB**	334	79.1
**Total**	422	100.0
	**n**_**2**_ = 88	
**EIB**_**WA**_	74	84.1
**EIB**_**A**_	14	15.9
**Total**	88	100.0

n_1_ – total number of study subjects, n_2_ – pupils diagnosed with EIB

*EIB*_*WA*_ EIB without asthma, *EIB*_*A*_ EIB with asthma

There was a significant difference in the mean post – exercise PEFR between study subjects with EIB versus no EIB and those with EIB_WA_ versus EIB_A_ – Table 8. Calculations were done with PEF readings obtained in the 5^th^ minute post exercise.

**Table 8:** Mean PEFR (L/min) of the pupils with EIB versus no EIB and EIB_WA_ versus EIB_A_ in the 5^th^ minute post—exercise

**Diagnosis**	**Frequency**	**Mean**	**Std. Dev**	**T**	***p*** **—value**	**Mean difference**	**95% CI of the difference**
**EIB**	88	187.39	56.31	- 7.69	* < 0.001	- 48.45	- 60.83 to—36.07
**No EIB**	334	235.84	51.53				
**EIB**_WA_	74	194.46	56.77	3.77	*0.01	44.46	20.24 – 68.68
**EIB**_A_	14	150.00	36.59				

t-test – test of association

*PEFR* Peak expiratory flow rate, *Std. Dev* Standard Deviation, *CI* Confidence interval

*Significant, *p* < 0.05

Most of the pupils with EIB_WA_/EIB_A_ (5^th^ minute) were of mild severity – Table 9.

**Table 9:** Severity of EIB_WA_ / EIB_A_ in the pupils

**EIB severity (5 min)** ^th^	**EIB (%)**_WA_	**EIB (%)**_A_	**Total (%)**
**Mild**	54 (85.7)	9 (14.3)	63 (100.0)
**Moderate**	20 (80.0)	5 (20.0)	25 (100.0)
**Severe**	_	_	_
**Total**	74 (84.1)	14 (15.9)	88 (100.0)

### Section D: Results on factors associated with EIB in the study subjects

This section provides results on factors associated with EIB and its subtypes in the study subjects. The section also gives results of logistic regression analysis between some of these factors and the diagnosis of EIB in the pupils.

The mean BMI for age and gender z scores of study subjects with EIB was—0.09 ± 1.09. Among the socio-demographic characteristics analysed, age, gender and the type of school attended (public or private) had significant associations respectively with the presence of EIB (5^th^minute) – Table 10.

**Table 10:** Association between socio-demographic characteristics and the presence of EIB in the study subjects

**Socio-demographic characteristics**	**EIB diagnosed (5** ^**th**^ ** min)**			**Total (%)**	**x** ^**2**^	***p*** ** – value**	**99% CI**
	Yes (%)	No (%)					
**Age (years)**							
** 6**	30 (22.6)		103 (77.4)	133(100.0)	30.65	* ** < 0.001**	**
** 7**	21 (25.3)		62 (74.7)	83(100.0)			
** 8**	12 (19.7)		49 (80.3)	61(100.0)			
** 9**	8 (10.8)		66 (89.2)	74(100.0)			
** 10**	7 (13.7)		44 (86.3)	51(100.0)			
** 11**	4 (28.6)		10 (71.4)	14(100.0)			
** 12**	6(100.0)		0 ( 0.0)	6(100.0)			
** Total**	88 (20.9)		334 (79.1)	334(100.0)			
**Gender**							
** Male**	64 (28.8)		158 (71.2)	222(100.0)	8.05	* ** < 0.001**	**
** Female**	24 (12.0)		176	200(100.0)			
** Total**	88 (20.9)		(88.0)	422(100.0)			
**SEC**							
** Upper**	51 (20.0)		204 (80.0)	255(100.0)	3.48	0.176	0.166 to
** Middle**	31 (25.6)		90 (74.4)	121(100.0)			0.185
** Lower**	6 (13.0)		40 (87.0)	46(100.0)			
** Total**	88 (20.9)		334 (79.1)	422(100.0)			
**BMI category**							
** Normal**	75 (23.7)		242(76.3)	317(100.0)	3.52	0.319	0.304 to 0.328
** Risk of overwt**	8 (16.3)		41(83.7)	49(100.0)			
** Overweight**	2 (25.0)		6(75.0)	8(100.0)			
** Wasted**	6 (12.5)		42(87.5)	48(100.0)			
** Total**	88 (21.9)		331(79.1)	422(100.0)			
**Type of school**							
** Public**	26 (29.5)		165(49.4)	191(100.0)	11.08	* **0.001**	0.000 to 0.001
** Private**	62 (70.5)		169(50.6)	231(100.0)			
** Total**	88 (20.9)		334(79.1)	422(100.0)			

Chi square (x^2^) – test of association

*Overwt* Overweight, *CI* Confidence interval

^*^Significant, *p* < 0.05. SEC – Socio-economic category

^**^ *p* values were so significant that confidence intervals could not be established (CI = 0.000 to 0.000)

SEC, BMI category and type of school had a significant association with categorization of pupils with EIB into those with EIB_WA_ / EIB_A_. All pupils who had EIB_A_ were from the upper class. All pupils found to be overweight had EIB_A_. Majority of the pupils with EIB_A_ were from private schools – Table 11.

**Table 11:** Association between some socio-demographic factors and EIB_WA_ / EIB_A_

Socio-demographic factors	EIB_WA_ (%)	EIB_A_ (%)	Total (%)	x^2^	*p*– value	99% CI
**SEC**						
**Upper**	37 (72.5)	14 (27.5)	51 (100.0)	12.08	* **0.005**	0.003 to 0.007
**Middle**	31(100.0)	0 (0.0)	31 (100.0)			
**Lower**	6 (100.0)	0 (0.0)	6 (100.0)			
**Total**	74 (84.1)	14 (15.9)	88 (100.0)			
**BMI category**						
**Normal**	60 (83.3)	12 (16.7)	72 (100.0)	13.25	* **0.015**	0.012 to 0.018
**Risk of overwt**	8 (100.0)	0 (0.0)	8 (100.0)			
**Overweight**	0 (0.0)	2 (100.0)	2 (100.0)			
**Wasted**	6 (100.0)	0 (0.0)	6 (100.0)			
**Total**	74 (84.1)	14 (15.9)	88 (100.0)			
**Type of school**						
**Public**	25 (96.2)	1 (3.8)	26 (100.0)	4.01	* **0.045**	
**Private**	49 (79.0)	13 (21.0)	62 100.0)			_
**Total**	74 (84.1)	14 (15.9)	88(100.0)			

Chi square (x^2^) – test of association

^*^Significant, *p* < 0.05. SEC – Socio-economic category. Overwt – Overweight. CI – Confidence interval

History of recurrent cough, history of recurrent wheeze, having a parent / guardian who smoked cigarette and having previously been diagnosed with asthma all had a significant association with the diagnosis of EIB with asthma (5^th^ minute) in the study subjects (*p* < 0.05) – Table 12. Out of the 20 pupils who had dry night cough, none had EIB_A_ and 3 (15%) had EIB_WA_ (*p* = 0.649).

**Table 12:** Association between respiratory features and EIB_A_ in the study subjects

**Respiratory features**	**Total (%)**	**EIB5** ^**th**^ **min (%)**_A_	**x** ^2^	***p*** **– value**	**99% CI**
**Recurrent Cough**	46 (100.0)	9 (19.6)	50.15	* < 0.001	**
**Recurrent Wheeze**	39 (100.0)	8 (20.5)	45.83	* < 0.001	**
**Parental/Guardian Cigarette smoking**	29 (100.0)	5 (17.2)	25.41	*0.014	0.011 to 0.017
**Previous diagnosis of Asthma**	17 (100.0)	5 (29.4)	39.63	*<0.001	**

Chi square (x^2^) – test of association

^*^Significant – *p* < 0.05. EIB_A_ – EIB with asthma

*CI* Confidence Interval

^**^ *p* values were so significant that confidence intervals could not be established (CI = 0.000 to 0.000)

Table 13 shows the association between features of allergy and the presence of EIB. History of allergic dermatitis and allergic rhinitis had a significant association to the presence of EIB (*p* = 0.001 and 0.033 respectively). While on examination, presence of reddish brown discolouration of the limbal conjunctivae as well as hyper-pigmentation of the antecubital fossae had significant associations with EIB (*p* =  < 0.001 and 0.028 respectively). EIB diagnosed in the 5^th^ minute post exercise was used for this analysis.

**Table 13:** Association between features of allergy and EIB in the 5^th^ minute post exercise

**Features of allergy**	**EIB diagnosed (5** ^**th**^ **min)**		**Total (%)**	**x** ^2^	***p*** **– value**
	**Yes (%)**	**No (%)**			
**History**					
**Allergic conjunctivitis**	12 (28.6)	30 (71.4)	42 (100.0)	1.68	0.194
**Allergic dermatitis**	14 (42.4)	19 (57.6)	33 (100.0)	10.09	*0.001
**Allergic rhinitis**	5 (9.6)	47 (90.4)	52 (100.0)	4.54	*0.033
**Physical examination**					
**Reddish/brown conjunctivae**	67 (27.5)	177 (72.5)	244(100.0)	15.30	* < 0.001
**Hyper-pig antecubital**	44 (17.3)	210 (82.7)	254(100.0)	4.82	*0.028
**Hyper-pig popliteal**	42 (20.1)	167 (79.9)	209(100.0)	0.14	0.704
**Hyper-pig neck**	58 (18.2)	261 (81.8)	319(100.0)	5.65	*0.017

Chi-square (x^2^) – test of association. *Significant, *p* < 0.05. Hyper-pig – Hyper-pigmentation

Logistic regression showed that a history of allergic conjunctivitis and allergic rhinitis were more likely to be found in children who had EIB (5^th^ minute) compared to reference values (odds ratio – 1.217, 5.832 respectively; *p* = 0.708, 0.001 respectively). On physical examination, the presence of reddish/ brownish discolouration of the limbal conjunctivae was approximately 83% less likely to be seen in children with EIB. Hyper-pigmentation of the antecubital fossae on the other hand was 2 times more likely in children with EIB and this was significant (OR – 2.740, p – 0.003). Details are given in Table 14. No child had rhonchi pre and post exercise.

**Table 14:** Relationship between features of allergy and presence of EIB (5^th^ minute) using logistic regression

**Allergic features**	**Odds ratio**	***p*** **– value**	**95% CI**
**History**			
**Allergic conjunctivitis**			
**No**	1.000		
**Yes (1)**	1.217	0.708	0.435 to 3.405
**Allergic dermatitis**			
**No**	1.000		
**Yes (1)**	0.161	* **0.001**	0.055 to 0.477
**Allergic rhinitis**			
**No**	1.000		
**Yes (1)**	5.832	* **0.001**	2.042 to 16.659
**Physical examination**			
**Reddish/brownish conjunctivae**			
**No**	1.000		
**Yes (1)**	0.186	* ** < 0.001**	0.097 to 0.358
**Hyper-pig antecubital**			
**No**	1.000		
**Yes (1)**	2.740	* **0.003**	1.410 to 5.323
**Hyper-pig popliteal**			
**No**	1.000		
**Yes (1)**	0.707	0.265	0.384 to 1.301
**Hyper-pig neck**			
**No**	1.000		
**Yes (1)**	2.635	* **0.003**	1.386 to 5.009

^*^Significant, *p* < 0.05

*Hyper-pig* Hyper-pigmentation, *CI* Confidence interval

## Discussion

The purpose of this study was to determine the presence of Exercise Induced Bronchospasm (EIB) and associated factors in primary school children in Nnewi, South-East Nigeria. The highest prevalence of EIB from this study was 20.9%. This implies that approximately 21 out of a 100 primary school children in Nnewi would manifest EIB. This is higher than the 9.2% reported by Kuti et al [14] in Ilesa, South-West Nigeria recently and 6% reported by Onazi et al [2] in North-West Nigeria. It is however closer to the estimated 20% given for school children in the latest update of the practice parameter by Weiler et al [7]. This high prevalence could be because EIB was sought for as an entity and not just as a diagnostic tool for asthma. Majority of the children with EIB were in the mild category and none was in the severe category. This might be favourable with regards to long term outcome and management.

EIB was observed in all the minutes post exercise however the highest prevalence occurred in the 5^th^ minute post exercise. The 5^th^ minute post exercise was also observed to have the lowest range of peak expiratory flow readings. This is similar to what was reported by Oviawe et al [17] while studying ventilatory response in children. They observed maximal bronchoconstriction by the 3^rd^ minute post exercise [17]. Kuti et al [14] also observed the highest prevalence of EIB in their study in the 5^th^ minute post exercise. This observation is probably due to exercise associated hyperpnoea. This hyperpnoea results in drying of the airways (which is the principal trigger for the cascade of events resulting in exercise induced bronchospasm) and is usually maximal few minutes after exercise. Subsequently, recovery sets in and respiratory rate falls.

There was a significant difference in the mean peak expiratory flow rate (PEFR) for those diagnosed with EIB versus those who did not have EIB. Majority of the study subjects with EIB had EIB_WA_. There was also a significant difference in the mean PEFR for those with EIB_WA_ versus those with EIB_A_. These differences were not observed in the mean pre exercise peak expiratory flow readings. This supports the latest recommendation that EIB is a clinical entity that can be diagnosed on its own and that among those who have EIB, they ought to be stratified based on the presence or absence of asthma [5–8]. The presence of EIB is not equivalent to the diagnosis of asthma [5–8].

Using the prevalence of EIB with asthma (EIB_A_) as a reflection of asthma prevalence, its prevalence was 3.3% in this study. This is lower than the prevalence of 6% reported by Onazi et al [2] in Gusau, North-West Nigeria. It is also lower than the asthma prevalence reported by the CDC [42]. It is however higher than the 0.7% reported by Oviawe [16] in a rural community in Southern Nigeria. This might point to a rising asthma prevalence as claimed [2, 43]. The increase in urbanisation in our environment has been identified to be responsible for this [43].

Majority of the study subjects with EIB_A_ were in the mild category. This could point to an adequate asthma control and possibly indicate a favourable prognosis [6, 7]. They all had normal BMI z scores and this might have also limited the severity of their EIB since excess weight did not increase their work of breathing. However, all the pupils found to be overweight had EIB_A_ and this supports the notion that increased weight has a negative influence on respiratory function [44, 45].

Respiratory features found to have a significant association with the diagnosis of EIB_A_ in the study subjects were recurrent cough, recurrent wheeze, having a parent/ guardian who smoked cigarette and a previous history of asthma. This is similar to what was reported by Onazi et al [2]. These respiratory features are in line with those which are part of the definition of asthma [9, 40].

Age had a significant association with the presence of EIB in the study subjects. EIB was found more in the younger age groups (6 – 8 years). This is in keeping with the finding made by N’gangaet al [11] in Kenya but is at variance with the report by Onazi et al [2] and Kuti et al [14]. This can be accounted for by the smaller size of the bronchioles in younger age which will result in a greater expression of bronchospasm [16]. In addition, younger children are known to have more frequent episodes of respiratory tract infections which can increase their predilection for bronchospasm following exercise [29]. Children have however been known to outgrow allergic tendencies and this might account for the reduction in involvement of older children.

More males (28.8%) were found to have EIB in this study and this was significant. This is similar to the report given by Onazi et al [2] among children in Gusau Northern Nigeria. This might be because the males are more likely to run more vigorously and as a result trigger airway spasm. Also, males are more playful especially in the school age and so are more likely to be exposed to factors that result in air way hyper-responsiveness.

Most of the children diagnosed with EIB were from upper social class. This is similar to what was reported by Ernst et al [46] in Canada. Majority of the study subjects in this research were from the upper and middle social classes. As a result, they may have been exposed to factors that resulted in abnormal immune response in early life which ultimately manifested as respiratory allergies. This is supported by the finding that all the pupils with EIB_A_ were exclusively from the upper class.

The mean BMI z scores for the study population was within normal and majority of the pupils diagnosed with EIB had normal values. This is not in line with the widely held opinion that obesity results in abnormal respiratory function [44, 45]. None of the study subjects was obese and only two of those diagnosed with EIB were overweight. This might explain why none of those with EIB fell into the severe range.

Most of the study subjects with EIB in this research were from private schools. Study subjects from the private schools contributed the greater proportion of upper (65.5%) and middle (50.4%) social classes. This does not tally with what was reported by Kuti et al [14] and Adewumi et al [15] in Nigeria. Children in higher social classes might have more exposures that trigger respiratory allergies such as bottle feeding and less outdoor activities. This is supported by the finding that the greater proportion of those with EIB_A_ were from private schools. On the other hand, children in public schools are known to walk to school and so might be more physically fit, having a greater amount of exercise tolerance. This can explain these findings.

Finally, among the other allergic features sought for, history of allergic dermatitis and allergic rhinitis as well as physical examination features suggestive of allergic dermatitis and allergic conjunctivitis were significantly associated to the diagnosis of EIB in the study subjects. These findings remained significant even after correcting for confounders using logistic regression. These findings are similar to those of other authors [2, 47, 48]. They can be explained by the fact that children with respiratory allergies have been known to show features of allergy in other systems [49].

## Conclusions

Exercise Induced Bronchospasm (EIB) in primary school children in Nnewi is high with the majority of the study subjects who had EIB having EIB_WA_ and being in the mild category. All pupils with EIB had normal nutritional status and majority with EIB were of high social class. Age and gender were significantly associated with the diagnosis of EIB. Features of allergic dermatitis, allergic conjunctivitis and allergic rhinitis were found in study subjects with EIB.

Awareness needs to be created among patients, parents/guardians and clinicians on the clinical entity—EIB as its presence does not equate asthma. This will facilitate proper diagnosis, documentation, management and prognostication.

## Data Availability

The datasets analysed during the current study are available from the corresponding author on reasonable request.

## Abbreviations

EIA: Exercise Induced Asthma
EIB: Exercise Induced Bronchospasm
EIB_A_: Exercise Induced Bronchospasm with Asthma
EIB_WA_: Exercise Induced Bronchospasm without Asthma
